# Cardiac tamponade associated with primary myelofibrosis: a case report

**DOI:** 10.1093/ehjcr/ytad630

**Published:** 2023-12-27

**Authors:** Wishnu Aditya Widodo, Teuku Muhammad Haykal Putra, Maria Elfiana, Eka Widya Khorinal

**Affiliations:** Department of Cardiology and Vascular Medicine, Jakarta Heart Center, Matraman Raya No.23, 13140 Jakarta, Indonesia; Department of Cardiology and Vascular Medicine, Jakarta Heart Center, Matraman Raya No.23, 13140 Jakarta, Indonesia; Department of Cardiology and Vascular Medicine, Jakarta Heart Center, Matraman Raya No.23, 13140 Jakarta, Indonesia; Department of Hemato-Oncology, Dharmais National Cancer Center, Jakarta, Indonesia

**Keywords:** Extramedullary haematopoiesis, Cardiac tamponade, Pericardial disease, Primary myelofibrosis, Constrictive pericarditis, Case report

## Abstract

**Background:**

Cardiac tamponade is a life-threatening condition that occurs when an abnormal amount of fluid accumulates in the pericardial sac and impedes the cardiac filling process. Although extremely rare, haematological diseases have the potential to trigger an extramedullary haematopoiesis (EMH) process within the pericardium, resulting in a substantial build-up of pericardial effusion.

**Case summary:**

We present the case of a 29-year-old male previously diagnosed with primary myelofibrosis (PMF), who presented to the emergency unit with cardiac tamponade. An emergent pericardiocentesis procedure was performed, successfully evacuating 850 mL of haemorrhagic fluid. Over the course of 3 days, a total of 1.5 L of haemorrhagic effusion were drained from the pericardial space. Analysis of the pericardial fluid revealed evidence of haematopoietic activity, suggesting a potential association with the EMH process occurring within the pericardium. Following a 7-day hospitalization, the patient was discharged in stable condition but later experienced the development of constrictive pericarditis.

**Discussion:**

Haemorrhagic pericardial effusion is a rare occurrence. The majority of cases stems from complications of medical procedures (iatrogenic), malignancies, or side effects of antiplatelet/anticoagulant medications. In patients with PMF, the impaired haematopoietic ability caused by the fibrotic process in the bone marrow compels the body to produce blood components elsewhere, a phenomenon known as EMH. On very rare occasions, EMH can develop in the pericardial space, potentially leading to life-threatening cardiac tamponade. Our patient was successfully managed through pericardial fluid evacuation and drainage but later developed constrictive pericarditis.

**Learning points**

- Exploring alternative causes of haemorrhagic pericardial effusion, which could lead to the development of cardiac tamponade.
- Understanding the pathophysiological processes by which haematologic diseases like primary myelofibrosis can give rise to extramedullary haematopoiesis (EMH), eventually culminating in pericardial effusion and life-threatening cardiac tamponade.
- Showing long-term cardiac consequence of a rare EMH process in the pericardium, which in our case leads to constrictive pericarditis.

## Introduction

Cardiac tamponade is a life-threatening medical emergency with a high mortality rate if not treated promptly and effectively. This condition arises from the accumulation of excessive fluid in the pericardial space and can have various underlying causes.^1–3^ In rare instances, haematological diseases can also contribute to the development of cardiac tamponade.^4,5^

Extramedullary haematopoiesis (EMH) represents an adaptive mechanism employed by the body to compensate for inadequacies in blood component production. In conditions like primary myelofibrosis (PMF), where the bone marrow becomes fibrotic, EMH is often observed. Although exceedingly rare, documented cases of EMH occurring within the pericardium emphasize the unique and diverse manifestations of this condition.^5–7^

We are reporting a case of cardiac tamponade with haemorrhagic pericardial effusion in a relatively young man who was already diagnosed with PMF. During our investigation, we discovered evidence of haematopoietic activity within his pericardium, raising suspicions of an EMH process occurring in this unusual location.

## Summary figure

**Table:** 

Day	Event
Day 0	Presented with severe respiratory distress, the patient was diagnosed with cardiac tamponade. Urgent pericardiocentesis and pericardial drain catheter placement. Patient had a pre-existing diagnosis of primary myelofibrosis and was on routine treatment with ruxolitinib 20 mg b.i.d.
Day 1	Pericardial fluid analysis revealed no evidence of tuberculosis or malignancy. Cytological examination indicated the presence of haematopoietic progenitor cell activity.
Day 3	The pericardial drain catheter was removed, and a total of 1500 mL of blood was drained from the pericardium over a 3-day period.
Day 7	Patient discharged from the hospital
Day 14	During clinical follow-up, the patient was found to have tachycardia and leg swelling. Echocardiography confirmed the presence of signs consistent with constrictive pericarditis.
Months 4–6	Ongoing tachycardia and persistent leg swelling were observed. Serial echocardiography exams indicated the presence of persistent constrictive pericarditis. The patient received routine colchicine treatment and continued taking his usual medication of ruxolitinib, along with weekly intravenous (IV) furosemide injections. Despite recommendations, the patient declined to undergo a pericardiectomy and was subsequently lost to follow-up.

## Case presentation

**Figure 1:**
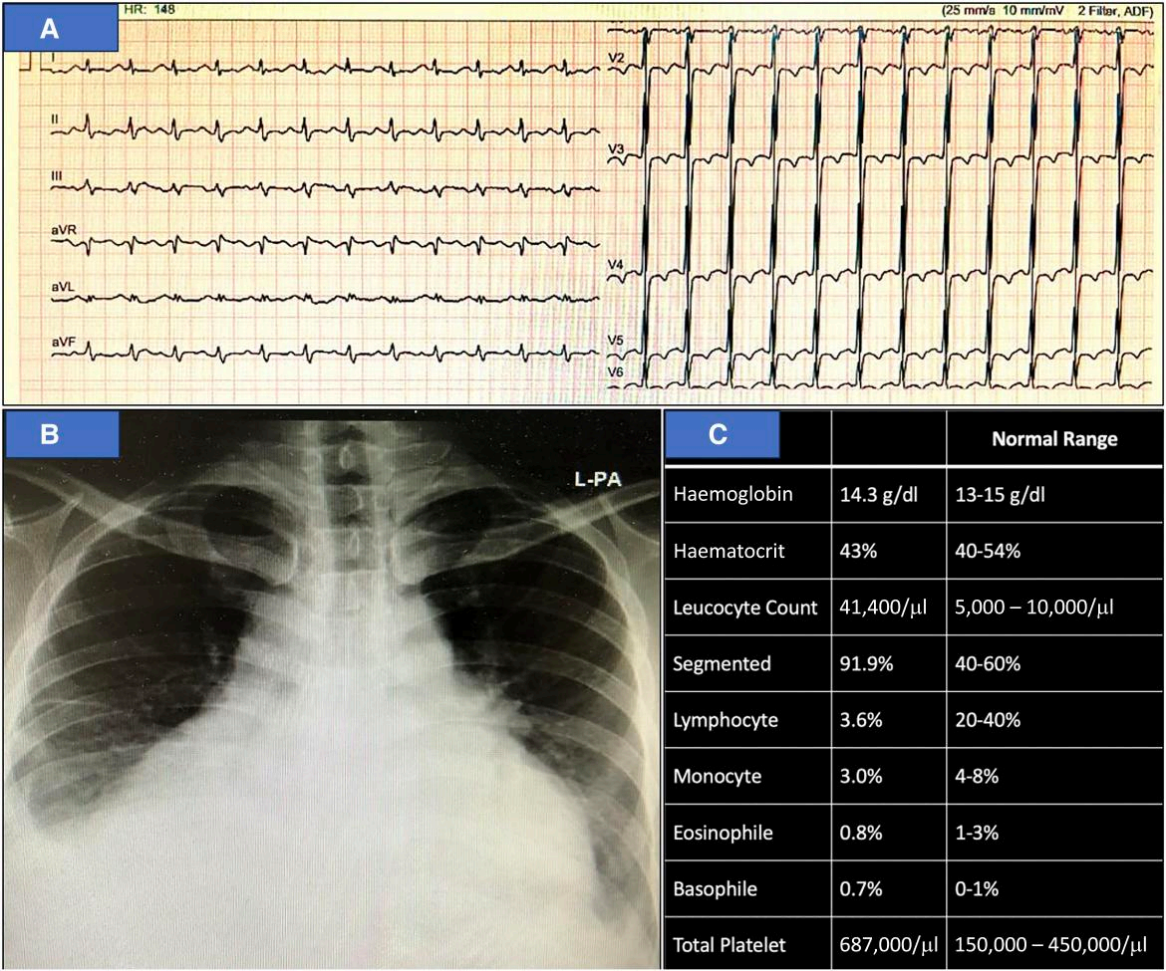
Diagnostic assessments during the initial emergency unit presentation. (*A*) Electrocardiography displaying sinus tachycardia. (*B*) Chest X-ray illustrating an enlarged cardiac silhouette. (*C*) Laboratory results indicating leucocytosis and thrombocytosis.

**Figure 2:**
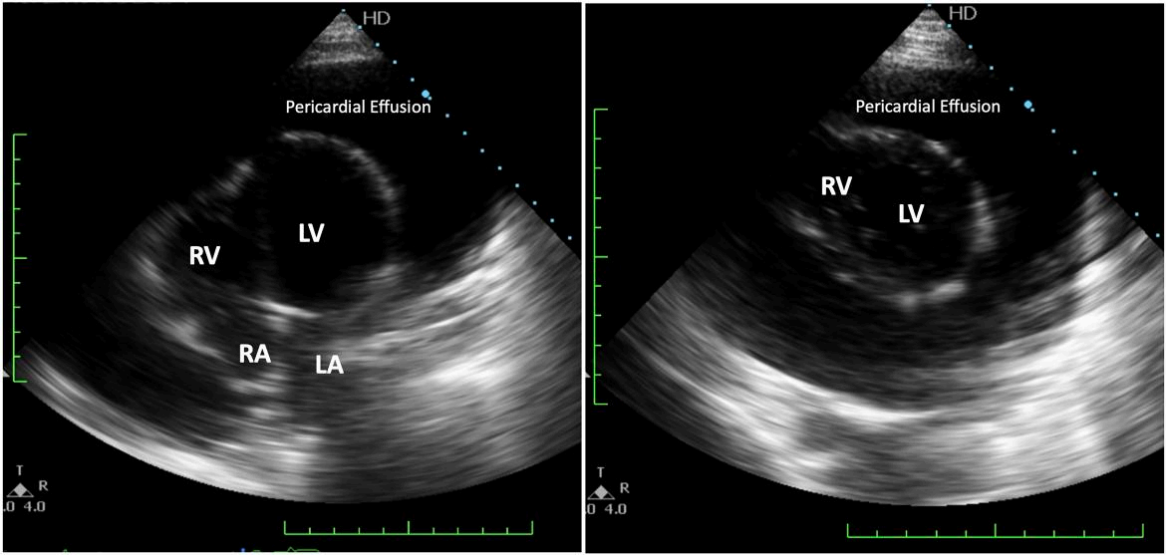
Echocardiogram from the initial emergency presentation, featuring views from the apical four-chamber and short-axis, displaying extensive pericardial fluid.

A 29-year-old male urgently presented to the emergency department due to severe breathlessness. On examination, his blood pressure was notably low at 90/50 mmHg, and he exhibited a rapid heart rate of 148 beats per minute (b.p.m.). Additionally, the patient displayed abdominal distension and significant enlargement of both the liver and spleen. An electrocardiogram revealed sinus tachycardia and relatively low voltage on limb leads (*Figure 1A*), along with an enlarged cardiac silhouette and bilateral pleural effusion evident on chest X-ray (*Figure 1B*). Laboratory results revealed leucocytosis and thrombocytosis, as the following: haemoglobin 14.3 g/dL (normal range 13–15 g/dL), leucocyte count 41,400/μL (normal range 5000–10,000/μL), and total platelet count of 687,000/μL (normal range 150,000–450,000/μL) (*Figure 1C*). Subsequent echocardiography disclosed a massive pericardial effusion, which led to a compressed right atrium (*Figure 2*). Of significant note, the patient had been diagnosed with PMF for the past year, with a confirmed Janus kinase 2 (JAK2) gene mutation. He had been undergoing routine treatment with a Janus kinase inhibitor ruxolitinib, taken at a dose of 20 mg twice daily for the preceding year.

**Figure 3:**
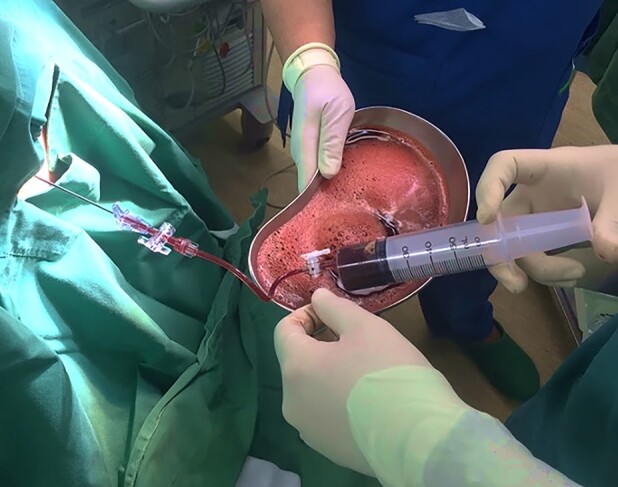
Initial pericardiocentesis fluid aspiration revealing haemorrhagic content.

**Figure 4:**
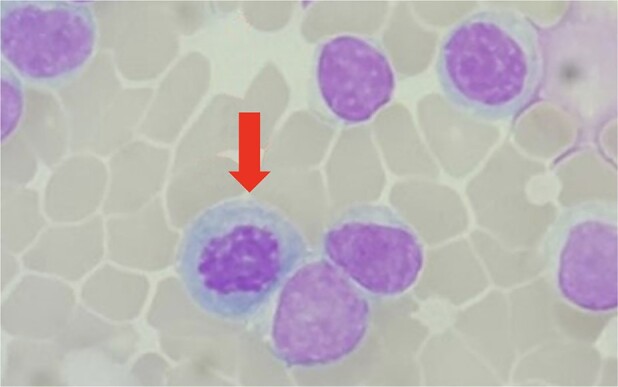
Cytomorphology evaluation showing immature myeloid cells with erythroid forms (leucoerythroblastic) displaying evidence of haematopoietic progenitor cell activity.

Emergency pericardiocentesis was performed immediately via subxiphoid access, with the insertion of a pigtail catheter for continuous drainage. Initially, the procedure removed 850 mL of haemorrhagic fluid (*Figure 3*). Over the 3-day hospitalization period, a total of 1500 mL of haemorrhagic fluid was evacuated. Subsequent fluid analysis ruled out tuberculosis and malignancy as causative factors. Cytomorphology evaluation of the fluid revealed the presence of nucleated red blood cells, neutrophil precursors, and immature myeloid cells with erythroid forms, indicating a leucoerythroblastic pattern and suggesting haematopoietic progenitor cell activity (*Figure 4*). Following this intervention and comprehensive care, the patient’s condition improved, and he was discharged in a stable condition after a 7-day hospital stay.

**Figure 5:**
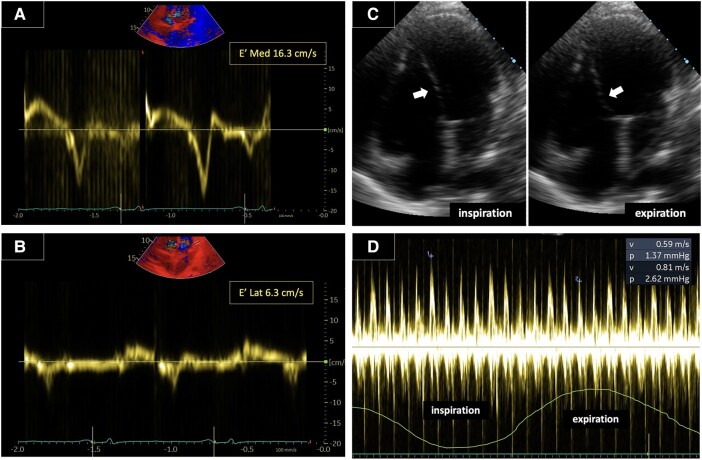
Echocardiogram during follow-up revealing signs indicative of constrictive pericarditis. (*A* and *B*) Tissue Doppler index (TDI) measurements demonstrating elevated E′ in the medial aspect and reduced E′ in the lateral aspect (annulus reversus). (*C*) Septal wall movement towards the left ventricle (LV) during inspiration and towards the right ventricle (RV) during the expiration phase, suggestive of further reduced LV filling during the inspiration phase (septal bounce/paradoxical septal movement). (*D*) Mitral inflow variation >25% with respiration, indicating significant fluctuations in mitral blood flow with breathing.

One week after discharge, the patient returned to the outpatient clinic in a stable condition but exhibited a relatively high resting heart rate ranging from 100 to 110 b.p.m. Clinical examination revealed bilateral leg oedema and ascites. A follow-up echocardiography examination demonstrated minimal pericardial effusion along with signs indicative of constrictive pericarditis, including paradoxical septal movement, increased mitral inflow variation >25%, and increased medial E′ compared with lateral E′ (annulus reversus) (*Figure 5*). These findings persisted throughout the 4-month follow-up period. Although a pericardiectomy was recommended as a potential treatment, the patient declined the procedure. Instead, he received weekly periodic intravenous furosemide injections to alleviate the oedema and ascites. In addition, his ruxolitinib 20 mg b.i.d treatment was continued, and colchicine was introduced at a dose of 0.5 mg twice daily in hopes of alleviating inflammation in the pericardium. However, after 6 months of outpatient visits, the patient did not return for further follow-up appointments.

Four years later, the author got in touch with the patient’s mother, who provided an update on the patient’s condition. She mentioned that he had chosen to discontinue all cardiac and haematologic medications since 3 years ago and had been steadily improving since then. During this period, he got married and became a father to a son. She shared recent photos that depicted him in good health, without any visible signs of an enlarged abdomen. She also mentioned that he had just celebrated his son’s first birthday. Despite being offered another complimentary medical follow-up, the patient declined to pursue it.

## Discussion

Primary myelofibrosis represents a clonal haematopoietic stem cell disorder characterized by chronic myeloproliferation and atypical megakaryocytic hyperplasia.^6^ A defining feature of PMF is bone marrow fibrosis (BMF), a consequence of non-clonal fibroblastic proliferation driven by abnormal growth factors released from clonally expanded megakaryocytes. Bone marrow fibrosis contributes to the impaired haematopoiesis that results in severe anaemia. Patients with PMF also commonly experience marked splenomegaly, EMH, and severe constitutional symptoms.^7–9^ Furthermore, PMF often presents with mutations in the JAK2, calreticulin (CALR), or myeloproliferative leukaemia (MPL) genes.^6^

Extramedullary haematopoiesis is a reactive process arising from bone marrow failure or ineffective circulation of mature blood elements. Common anatomical sites for EMH include the paraspinal thoracic region, liver, and spleen. Uncommonly, EMH can manifest in renal, mesenteric, pericardial, and pleural spaces.^5^ The mechanisms underlying EMH in PMF remain incompletely understood but may involve abnormal marrow precursor release into circulation, as seen in animal models.^10–12^ Increased trafficking of CD34+ cells into circulation may also contribute.^13^ Extramedullary haematopoiesis, however, tends to be less efficient than medullary haematopoiesis, often resulting in the cytopenia frequently observed in this condition. In our case, the patient had a confirmed diagnosis of PMF characterized by elevated red blood cell, leucocyte, and platelet counts (polycythaemia) alongside splenomegaly, indicative of inadequate haematologic function compensated by alternative mechanisms.

Pericardial effusion refers to fluid accumulation in the pericardial space, with the potential to induce cardiac tamponade when pressure exceeds the normal range, disrupting cardiac filling.^2^ Pericardial effusions can manifest as transudative, exudative, sanguineous, or haemorrhagic, contingent upon the underlying cause.^3^ Haemorrhagic pericardial effusions may result from procedural complications (iatrogenic), malignancies, or as an adverse effect of antiplatelet/anticoagulant drugs.^4^ In our patient’s case, there was no history of thoracic interventions or surgical procedures, chest trauma, or antiplatelet/anticoagulant medication use. Pericardial fluid analysis revealed no malignant cells but did indicate the presence of nucleated red blood cells, neutrophil precursors, and immature myeloid cells with erythroid forms, suggesting haematopoietic activity in the pericardial cavity. Given the underlying PMF condition with splenomegaly and polycythaemia, along with evidence of haematopoietic activity in the pericardial fluid, it strongly suggests the occurrence of EMH within the pericardial space—a rare and scarcely reported phenomenon.^4,5,7^ The mechanisms driving EMH are not fully understood, and the emergence of EMH activity in the pericardial space is unpredictable.^5,7^

Neoplastic pericardial disease may lead to constrictive pericarditis in an average of 2–5% of cases.^1^ In our patient, this diagnosis was established based on typical clinical manifestations (breathlessness, tachycardia, ascites, oedema) and characteristic echocardiography findings (septal bounce, increased mitral inflow variation, and annulus reversus).^2,14^ Constrictive pericarditis in patients with EMH may result from changes in pericardial structure, with infiltration by megakaryocytes, immature erythroid, and granulocytic cells.^15^ The informal follow-up provided by the patient’s mother raised intriguing questions about the potential for spontaneous resolution of constrictive pericarditis. To date, there are no reports on such a scenario, and we hope for an opportunity to conduct further clinical follow-up on this patient in the future.

## Supplementary Material

ytad630_Supplementary_Data

## Data Availability

The data underlying this article are available in the article and in its online Supplementary material.

## Lead author biography

Wishnu Aditya Widodo is a cardiologist who currently serves as the Head of the Cardiology Department at Jakarta Heart Center in Indonesia. He completed his cardiology training at Harapan Kita Hospital, Indonesia, and furthered his expertise through a fellowship in interventional cardiology at the National Heart Center in Singapore. His daily routine includes performing clinical duties, cardiac interventions, and research as well as training junior staff.
